# Dr. Bradley on Puerperal Fever

**Published:** 1812-03

**Authors:** James Bradley

**Affiliations:** Huddersfield


*To the Editors of the Medical and Physical Journal.*

GENTLEMEN,

IT is only within these few days that I have, for the first time, seen Dr. Ramsbotham's communication on Puerperal Fever, in your Journal for October last, who is laudably anxious to arrest the progress of this fatal malady, which has lately threatened the populous city of London. On this paper, I beg leave to offer a few candid remarks, having previously called his attention to two cases I published in your Journal for last March. In one of these, large doses of calomel, in conjunction with other purgatives, were given with the happiest effects. In the other case, smaller doses of this mineral, combined with opium, was administered with similar success. I would not have obtruded these cases on Dr. Ramsbotham's attention, was I not convinced that every useful hint relating to a complaint that has hitherto so much baffled the skill of the first practitioners in this county, cannot be too generally known.

The principal value of this gentleman's communication consists, in my opinion, in the description he gives us of the appearances on dissection, and in recommending copious bleeding, in conformity with the practice of Dr. Gordon, a name that cannot be too familiar to every obstetric practitioner; but, notwithstanding what he says respecting phlebotomy, which, in the main, is correct, and consonant to the best observation, yet he does not *particularly* tell us under what circumstances this operation is or is not adviseable. What Mr. Hey says respecting copious bleeding being only admissible on the first day of the attack, is certainly judicious, (meaning, no doubt, if we bleed at all, it must be within this period;) but, as it not unfrequently happens that this complaint runs through all its different stages in twenty-four hours, and in some of these bleeding would be decidedly improper, it follows, that Mr. Hey's position is too general and distant to bear directly on the question.

Dr. Ramsbotham, having very properly urged the necessity of early blood-letting, adds, "If considerable symptoms of irritation and debility have shewn themselves, or if effusion into the abdominal cavity has taken place, such treatment must necessarily be unsuccessful, and get into disgrace." What are these considerable symptoms of irritation and debility? Now, as there are so many intermediate stages betwixt high inflammatory action, and the last stage of the complaint, where there is excessive debility, I will ask, at

 what what point does the former cease, and the latter begin. One practitioner will answer one thing, a second another. Hence, that confusion and uncertainty, which always will attend the want of appropriate and definite terms. I will beg leave to recommend to Dr. Ramsbotham something like a test, or criterion for determining on the propriety of blood-letting, in this dangerous affection. Such may not be unworthy of his attention, till some more experienced practitioner can better supply this *desideratum*.

There are no other symptoms peculiar to this affection, that exhibit such a variety of appearances as the tongue, when the complaint runs through all its different stages. For practical purposes, I shall divide these appearances into three different stadia, being confident it can only be by such a measure that I can render myself tolerably understood. It must, however, be observed, that these divisions comprehend many intermediate shades, which would be too tedious and difficult to describe.

The first of these stages is, when the tongue is white, which is sometimes observable at the first onset of the attack, or, what is oftener the case, partly white and partly yellow, the latter tinge being confined more especially to the middle part of it. The second is, when the whole tongue puts on a deeply lemon or orange colored appearance, and the fur is of more considerable thickness. The third and last stage is, when the incrustation is from a light brown to a sooty color, and often parched.

It is only in the first of these stages that phlebotomy can be liberally practised with advantage, and it will be necessary to observe that the quantity of blood taken must always, *cæteris paribus*, be in an inverse ratio to the yellowness of the tongue. I have seen two cases of the second description, where, from the state of the pulse, as well as great tightness and irritation in the chest, bleeding seemed indispensible; but, on taking away ten ounces of blood in the one case, and twelve in the other, the pulse was rendered smaller, more tremulous, and, in point of velocity, accelerated. Delirium almost immediately ensued, especially in one of the cases, and the change was so sudden as forcibly to impress the minds of their friends with an idea that bleeding had destroyed them.

The state of the pulse, abstractedly, is seldom a criterion for determining on this operation, in any stage of the disorder; for, in these two cases, the pulse assumed more action and tension than usually occur in this complaint; but the pain was principally in the right hypochondrium, and, in one of the instances, shot towards the scrobiculus cordis, and was uncommonly acute.

I think

I think observation warrants me saying, that bleeding, in the first stage, tends to diminish, or at least to arrest, the increasing velocity of the pulse; but, in the second stage, or under any other circumstance of the complaint, it will increase the number of vibrations, and consequently do harm.

Dr. Ramsbotham says, 'the symptoms preceding recovery, in this complaint, are a diminution in the velocity of the pulse, of the pain and tension of the abdomen, refreshing sleep, and a change of posture,' and, he might have added, the re-appearance of the mammary, and cutaneous secretions, with a return of the lochia, either in part or *in toto*, as well as an increase of urine. It was on the principle of restoring these, that calomel, combined with opium, was administered in Mr. North's case, (being one of those above alluded to) on a supposition *à priori*, that the former was as likely to promote glandular secretion, as well as a return of the lochial discharge; and that the latter would determine to the skin, as well as abate irritation. The effects more than answered expectation, but, as to the *modus agendi* of this mineral, or whether it acted mechanically or otherwise, as a specific, I cannot say; it however arrested the progress of the complaint, both when given as a purgative and an alterant, not only by amending the state of the tongue, and diminishing irritation, but in increasing or restoring the suspended secretions. The practice, however, of liberally purging with calomel, ought, in my opinion, to be confined to the first stage of the disorder; and, at a more advanced period, smaller doses, combined with opium, may be preferable, copious blood-letting being always premised, as often as that operation can be seasonably performed.

The impropriety of bleeding, when effusion has taken place in the abdomen, is a position that will be universally admitted; but what are the symptoms by which this event can be ascertained? Judging partly from observation, and partly from analogy, I should, when the pulse has less of a cordy feel, and rather more contracted, when there is some remission of the pain, preceded by, or sometimes attended with, rigors, and generally succeeded by tumefaction of the abdomen, together with increased languor and dejection, and when the fur of the tongue seems rather cracked and looser, then conclude that either effusion or suppuration had taken place. Symptoms analogous to these, especially as far as relate to the appearance of the tongue, we know accompany suppuration, which forms extensively beneath any membranous covering, previously in a state of active inflammation, and more particularly when confined to hte parietes of the abdomen.

The loose desquamatory appearance of the incrustation on the tongue is strongly characteristic of effusion or suppuration; and, when taken in conjunction with the other symptoms above described, is generally conclusive. Under these circumstances, the tongue seldom displays that hue which is peculiar to the last stage of the complaint; but, on the other hand, is of an orange colour; and, on the whole, rather calculated to inspire some hopes of amendment in the patient's case: but these appearances are fallacious, as the disorder continues insidiously to undermine the powers of life, as the event soon demonstrates.

Dr. Ramsbotham also maintains, that there are some cases that seem determinedly fatal from the first onset. What are the phenomena peculiar to these cases? Why has he not pointed out the specific difference in the diagnosis of this variety? Might not this observation be extended to all other dangerous disorders, where the operation of constitutional causes and medical treatment influence the event?

I do not wish to enter into any farther disquisition on any speculative opinions this gentleman may have advanced. One exception, however, and which might be acted upon, and is neither the result of observation nor experience, and by being fraught with practical consequences, I beg leave to make. I allude to what he says, respecting the use of the digitalis in this complaint; and, as his reasons for recommending it are neither founded on facts nor analogy, they ought to be received with becoming caution. On the other hand, if he had administered this medicine in a few cases with advantage, and detailed them faithfully to the public, the profession would have received them gratefully. In what affection of a typhoid nature, has the digitalis proved useful? In the *pneumonia typhoides*, which bears some similarity to this affection, would not most physicians consider the digitalis a very doubtful remedy, though better calculated *à priori* for the former than the latter? But Dr. Ramsbotham may say, that what he has advanced respecting this remedy is only matter of opinion; but all teachers of, or lecturers in, any branch of medical science, ought to be aware that such is often authority, especially in the minds of their juvenile hearers, whose judgment in future life is not unfrequently too much warped by scholastic prejudice.

On such authority, also, incalculable mischief might attend the administration of an active, powerful, and *untried*, medicine, in a dangerous disorder, where the thread of life is soon divided, before it fell into general disuse; for it is well known, that whatever is once adopted in practice will have have its season of reputation. Had he restricted the administration of this medicine to the removal of effusion only, he would have adduced something like analogy to his support; for, perhaps, no remedy has yet been found equal to the digitalis, in obviating effusion consequent to some kinds of inflammation, and pneumonic in particular; but the character of puerperal is so essentially different from inflammation in general, as to admit of no parallel; and, besides, at this period of the disorder, the animal powers are nearly exhausted, insomuch, that the patient often survives not many hours. Under such circumstances, few practitioners would think the digitalis adviseable; for, generally to insure any advantage from its use, a tolerable share of remaining strength and vigor is required.

I am, &c.

JAMES BRADLEY.

*Huddersfield, Dec.* 20, 1811.

